# Lights and Shadows in the Genetics of Syndromic and Non-Syndromic Hearing Loss in the Italian Population

**DOI:** 10.3390/genes11111237

**Published:** 2020-10-22

**Authors:** Anna Morgan, Stefania Lenarduzzi, Beatrice Spedicati, Elisabetta Cattaruzzi, Flora Maria Murru, Giulia Pelliccione, Daniela Mazzà, Marcella Zollino, Claudio Graziano, Umberto Ambrosetti, Marco Seri, Flavio Faletra, Giorgia Girotto

**Affiliations:** 1 Institute for Maternal and Child Health–IRCCS “Burlo Garofolo”, 34137 Trieste, Italy; stefania.lenarduzzi@burlo.trieste.it (S.L.); beatrice.spedicati@burlo.trieste.it (B.S.); elisabetta.cattaruzzi@burlo.trieste.it (E.C.); floramaria.murru@burlo.trieste.it (F.M.M.); giulia.pelliccione@burlo.trieste.it (G.P.); daniela.mazza@burlo.trieste.it (D.M.); flavio.faletra@burlo.trieste.it (F.F.); giorgia.girotto@burlo.trieste.it (G.G.); 2 Department of Medicine, Surgery and Health Sciences, University of Trieste, 34125 Trieste, Italy; 3 Fondazione Policlinico Universitario A. Gemelli, IRCCS, UOC Genetica, 00168 Rome, Italy; Marcella.Zollino@Unicatt.it; 4 Istituto di Medicina Genomica, Università Cattolica Sacro Cuore, 00168 Rome, Italy; 5 Unit of Medical Genetics, S. Orsola-Malpighi Hospital, 40138 Bologna, Italy; claudio.graziano@unibo.it (C.G.); marco.seri@unibo.it (M.S.); 6 Audiology and audiophonology, University of Milano/Fondazione IRCCS Cà Granda Ospedale Maggiore Policlinico, 20122 Milano, Italy; umberto.ambrosetti@unimi.it

**Keywords:** hereditary hearing loss, MLPA, whole exome sequencing, molecular diagnosis

## Abstract

Hearing loss (HL), both syndromic (SHL) and non-syndromic (NSHL), is the most common sensory disorder, affecting ~460 million people worldwide. More than 50% of the congenital/childhood cases are attributable to genetic causes, highlighting the importance of genetic testing in this class of disorders. Here we applied a multi-step strategy for the molecular diagnosis of HL in 125 patients, which included: (1) an accurate clinical evaluation, (2) the analysis of *GJB2, GJB6,* and *MT-RNR1* genes, (3) the evaluation *STRC-CATSPER2* and *OTOA* deletions via Multiplex Ligation Probe Amplification (MLPA), (4) Whole Exome Sequencing (WES) in patients negative to steps 2 and 3. Our approach led to the characterization of 50% of the NSHL cases, confirming both the relevant role of the *GJB2* (20% of cases) and *STRC* deletions (6% of cases), and the high genetic heterogeneity of NSHL. Moreover, due to the genetic findings, 4% of apparent NSHL patients have been re-diagnosed as SHL. Finally, WES characterized 86% of SHL patients, supporting the role of already know disease-genes. Overall, our approach proved to be efficient in identifying the molecular cause of HL, providing essential information for the patients’ future management.

## 1. Introduction

Hereditary Hearing Loss (HHL) is the most common sensory disorder in childhood and adulthood, affecting approximately 1–3 out of 1000 newborns [1].

Genetic factors account for more than 50% of all the cases, where the majority exhibit an autosomal recessive (AR) pattern of inheritance (75–80%) followed by 20–25% of autosomal dominant (AD) cases and 1–1.5% of X-linked or mitochondrial ones [2].

More than 200 genes (i.e., ~1% of all the coding genes) are involved in the hearing process [3]; therefore, it is not surprising that HHL displays substantial genetic heterogeneity. The many genetic forms of hearing loss can be further categorized into syndromic and non-syndromic conditions, which, respectively, constitute 30% and 70% of the genetic causes of congenital HHL, with syndromic HHL likely underestimated [4,5]. To date, about 170 loci and 117 genes (36 autosomal dominant (AD), 65 autosomal recessives (AR), 11 AD/AR, and 5 X-linked genes) have been reported as causative of Non-Syndromic Hearing Loss (NSHL) (Hereditary Hearing Loss Homepage; http://hereditaryhearingloss.org/), and more than 400 syndromes associated with hearing loss and other symptoms (Syndromic Hearing Loss—SHL) have been described [6]. In particular, among the syndromes identified so far, some of them appear more frequently than the others (e.g., Usher syndrome compared to Waardenburg syndrome) [7,8]), although, in some cases, the full spectrum of clinical features might be subtle, or even not present until later in life [9].

The implementation of next-generation sequencing technologies (NGS), together with molecular karyotyping (e.g., Single Nucleotide Polymorphism (SNP) array or Comparative Genomic Hybridization (CGH) array) and other validating assays (e.g., Multiplex Ligation Probe Amplification—MLPA), has dramatically increased the diagnostic rate of HHL, leading to the identification of several mutations and Copy Number Variations (CNVs) in known deafness genes, as well as to the discovery of new disease genes [10,11,12,13]. The possibility to simultaneously screen large number of genes is essential to address with the genetic heterogeneity of HHL. This aspect is fundamental for NSHL, where, apart from the relevant contribution of the *GJB2* gene and, in some populations of *GJB6* gene, both responsible for ~50% of all AR cases [14,15,16], and of *STRC* deletions (accounting for 1% to 5% of HL cases [17]), no other worldwide primary players have been identified.

**Figure 1:**
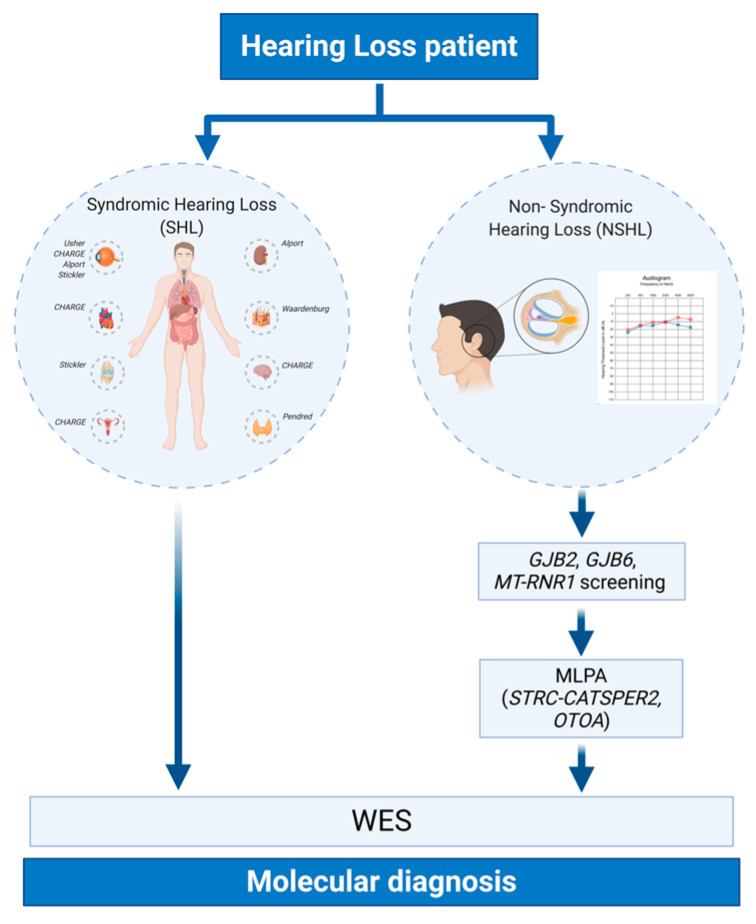
Schematic representation of the multi-step strategy applied for the study of Hereditary Hearing Loss (HHL). All of the patients enrolled in the present study underwent a careful clinical examination to distinguish between Syndromic Hearing Loss (SHL) and Non-Syndromic Hearing Loss (NSHL). Afterwards, NSHL patients were screened for mutation in *GJB2*, *GJB6,* and *MT-RNR1* genes, and for deletions in *STRC-CATSPER2* and *OTOA* genes. All of the NSHL patients negative to the first-level screening, together with SHL patients, have been then analyzed through Whole Exome Sequencing (WES).

In the present work, we applied a multi-step strategy to identify the genetic cause of HHL in a subset of 125 individuals recruited in the last two years. The protocol included: (1) an accurate clinical evaluation of all the patients and their relatives to exclude all the cases in which HL was due to non-genetic causes (i.e., middle ear anomalies, infections, ototoxic drugs, etc.) and to distinguish between NSHL and SHL; (2) the analysis of *GJB2*, *GJB6* and *MT-RNR1* genes in patients affected by NSHL; (3) the evaluation of *STRC-CATSPER2* and *OTOA* deletions in case of negativity to step (2); (4) Whole Exome Sequencing (WES) analysis in case of negativity to steps (2) and (3) and for patients affected by SHL (Figure 1).

The present work results illustrate the genetic heterogeneity of HHL and the importance of a detailed clinical characterization combined with high-throughput technologies for the diagnosis of both NSHL and SHL.

## 2. Materials and Methods

### 2.1. Ethical Statement

All patients signed written informed consent forms for both genetic counseling and molecular genetic testing. In the case of minors, informed consent was obtained from the next of kin. The study was approved by the Institutional Review Board (IRB) of the Institute for Maternal and Child Health (IRCCS) Burlo Garofolo, Trieste, Italy. All research was conducted according to the ethical standards as defined by the Helsinki Declaration.

### 2.2. Clinical Evaluation and Sample Collection

A total of 125 HHL patients have been recruited in the following centers (Otorhinolaryngology or Medical Genetics): Trieste (IRCCS Burlo Garofolo), Milano (IRCCS Cà Granda—Ospedale Maggiore Policlinico), Rome (Policlinico Universitario "A. Gemelli"), and Bologna (Policlinico S. Orsola-Malpighi).

All participants underwent pure tone audiometric testing (PTA) or auditory brainstem response (ABR) in order to characterize the severity of HL according to the international guidelines described by Clark (1981) [18]. Moreover, neurological and ophthalmological examinations, electrocardiogram, kidney ultrasonography, Magnetic Resonance Imaging (MRI) and Computerized Tomography (CT) scan, and thyroid function were carried out on routine basis in all probands.

Based on the clinical findings, 118 patients were classified as NSHL and seven as SHL. Furthermore, depending on the pedigree structure, the cases were divided into sporadic (*n* = 90) and familial (*n* = 35), the latter being classified as likely autosomal recessive (AR) (*n* = 9), and likely autosomal dominant (AD) (*n* = 26).

### 2.3. GJB2, GJB6, and mtDNA Analysis

For all the patients, the entire coding region of *GJB2* was analyzed by Sanger sequencing (primers available upon request). According to the manufacturer’s instructions, DNA was sequenced on a 3500 Dx Genetic Analyzer (Life-Technologies, Carlsbad, CA, USA), using ABI PRISM 3.1 Big Dye terminator chemistry (Life Technologies, Carlsbad, CA, USA). Moreover, *GJB6* deletions (D13S1830-D13S1854) were screened by multiplex PCR using the method described by del Castillo et al. 2002 [19], while The A1555G mtDNA mutation was tested by Restriction Fragment Length Polymorphism (PCR-RFLP) analysis using BsmAI as restriction enzyme, followed by visualization on an agarose gel stained with ethidium bromide.

### 2.4. Multiplex Ligation Probe Amplification (MLPA)

MLPA analysis for identification of deletion/duplication in *STRC*-*CATSPER2* and *OTOA* genes was conducted using SALSA^®^ MLPA^®^ probe mixes P461-A1 DIS (MRC-Holland, Amsterdam, the Netherlands) according to the manufacturer’s instructions. In brief, 50–100 ng DNA was denatured and hybridized overnight at 60 °C with the SALSA^®^ probe mix. Samples were then treated with DNA ligase for 15 min at 54 °C. The reaction was stopped by incubation at 98 °C for 5 min. Finally, PCR amplification was carried out with specific fluorescent-labeled PCR primers. After amplification, the amplified products’ fragment analysis was performed on ABI 3500dx Genetic Analyzer (Life-Technologies, Carlsbad, CA, USA).

Coffalyser.Net software was employed for data analysis in combination with the lot-specific MLPA Coffalyser sheet. The dosage quotient (DQ) of the reference probes in the patient samples was between 0.80 and 1.20. The following cutoff values for the DQ of the probes were used to interpret MLPA results; 0.80 < DQ < 1.20 (no deletion/duplication), DQ  =  0 (deletion), and 1.75 < DQ < 2.15 (duplication).

### 2.5. Whole Exome Sequencing (WES)

WES was completed on an Illumina NextSeq 550 instrument (Illumina, San Diego, CA, USA) with NextEra Flex for enrichment–Exome panel reagents, according to the manufacturer’s protocol.

Secondary analysis has been carried out using Isis Software (v.2.5.42.5-Illumina, Illumina, San Diego, CA, USA), i.e., reads alignment with Burrows-Wheeler Aligner (BWA) 0.7.7-isis-1.0.2 and variant calling with Isaac Variant Caller v. 2.1.4.2.

Single Nucleotides Variations (SNVs) and small Insertions and Deletions (INDELs) were collected into a standardized Variant Call Format (VCF) version 4.1 [20]. SNVs and INDELS were then annotated with ANNOVAR [21] using human genome build 19 (hg19) as the reference.

SNVs leading to synonymous amino acids substitutions not predicted as damaging, not affecting splicing or highly conserved residues were excluded, as well as SNVs/INDELs with quality score (QUAL) < 20 and called in off-target regions.

A comparison between the identified genetic variants and data reported in NCBI dbSNP build153 (http://www.ncbi.nlm.nih.gov/SNP/) as well as in gnomAD (http://gnomad.broadinstitute.org/), and National Heart, Lung and Blood Institute (NHLBI) Exome Sequencing Project (ESP) Exome Variant Server (Exome Variant Server, NHLBI GO Exome Sequencing Project (ESP), Seattle, WA) led to the exclusion of those variants previously reported as polymorphism. In particular, a Minor Allele Frequency (MAF) cutoff of 0.005 for recessive forms and 0.001 for the dominant ones was used.

The pathogenicity of known genetic variants was evaluated using ClinVar (http://www.ncbi.nlm.nih.gov/clinvar/), Deafness Variation Database (http://deafnessvariationdatabase.org/), and The Human Gene Mutation Database (http://www.hgmd.cf.ac.uk/ac/index.php).

Several in silico tools, such as PolyPhen-2 [22], Sorting Intolerant from Toleran (SIFT) [23], MutationTaster [24], Likelihood Ratio Test (LRT) [25], and Combined Annotation Dependent Depletion (CADD) score [26] were used to evaluate the pathogenicity of novel variants. Moreover, the evolutionary conservation of residues across species was evaluated by phyloP [27] and Genomic Evolutionary Rate Profiling (GERP) [28] scores.

Human Splicing Finder (HSF) version 2.4.1 [29] and Splice Site Prediction by Neural Network (NNSPLICE) version 9 (www.fruitfly.org) were adopted to predict the effect of the splice site mutations.

Finally, on a patient by patient basis, variants were discussed in the context of phenotypic data at interdisciplinary meetings and the most likely disease-causing SNVs/INDELs were analyzed by direct Sanger sequencing.

Sanger sequencing was also employed to perform the segregation analysis within the Family.

## 3. Results

During the last two years, 125 patients have been tested for *GJB2*, *GJB6,* and the A1555G mitochondrial mutation. Twenty percent of them were positive for mutations in the *GJB2* gene (i.e., 25 patients) with the c.35delG being the most frequent mutation (i.e., 44% of patients c.35delG homozygotes and 48% c.35delG carriers, together with other *in-trans* mutations, such as the p.(Glu120del), p.(Trp24 *) and p.(Glu47 *)) (Table 1). None of them carried deletions in *GJB6* or the A1555G mutation in the mitochondrial gene *MT-RNR1*.

**Figure 2:**
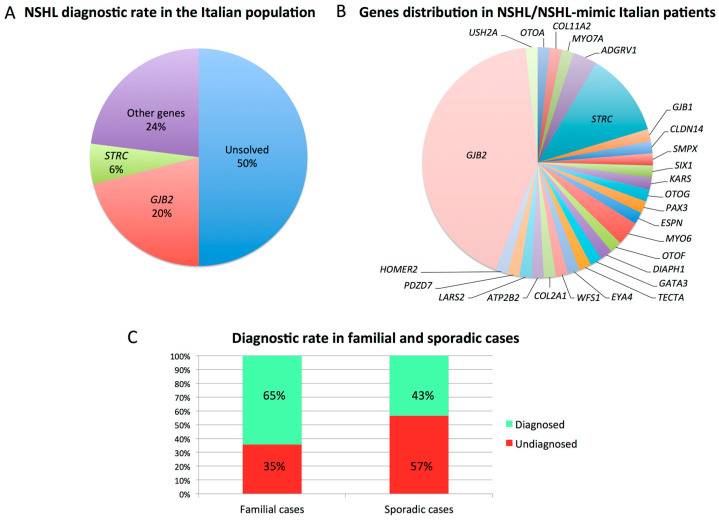
Diagnostic rate and genes distribution in NSHL patients. (**A**) Overall diagnostic rate for NSHL. Moreover, 50% of patients received a conclusive molecular diagnosis, with *GJB2* being the most frequently mutated gene (i.e., 20%), followed by *STRC* (i.e., 6%). (**B**) Genes distribution among all the NSHL and NSHL-mimic patients investigated. (**C**) Diagnostic rate comparison between familial and sporadic cases showing a higher percentage of solved cases among patients presenting with a familial history of HL.

Ninety-three NSHL individuals negative to this first-level screening were analyzed through MLPA to search for *STRC-CATSPER2* and *OTOA* deletions. Overall 8% of cases carried a homozygous deletion in *STRC* (*n* = 6) or *OTOA* (*n* = 1) genes (Table 2). The remaining 86 patients, together with their relatives, were then analyzed using WES. Sequencing data analysis led to the molecular diagnosis in 26 additional patients (Table 2) reaching an overall detection rate (i.e., *GJB2*/*GJB6*/mtDNA screening, MLPA, WES) of 50% (Figure 2A,B).

In particular, 65% of familial cases (i.e., 22/34) were genetically characterized, while for sporadic cases, the molecular cause was identified in 43% of patients (i.e., 36/84) (Figure 2C).

WES data allowed unveiling some peculiar scenarios, which reflect the complexity of NSHL.

In particular, WES allowed: (1) to detect syndromes in patients displaying only subtle phenotypic features; (2) to early diagnose diseases with a late-onset clinical manifestations; (3) to identify mutations in more than one gene involved in the same phenotype; (4) to molecularly characterize multiple genetic conditions in the same patient; and (5) to clarify the role of recently discovered genes.

An example of 1) is Family 10, who came to genetic counseling with a clinical diagnosis of NSHL in the proband and in the mother. WES revealed the presence of a novel heterozygous variant in *PAX3* (NM_181457.3) (c.220C > T p.(Arg74Cys)), which occurred as de novo in the mother and was inherited from the proband. *PAX3* is a gene known for being causative of Waardenburg syndrome type 1 and 2 [47], a disease characterized by HL, pigmentation abnormalities and, in some cases, dystopia canthorum or other additional features [8]. A clinical re-evaluation of the patients revealed the presence of mild pigmentary disturbances of the iris, hair, and skin, confirming the molecular diagnosis.

Regarding point 2), three patients who only displayed sensorineural hearing loss have been molecularly classified as Usher patients. In particular, two of them carried pathogenic mutations in the *GPR98* gene (NM_032119.3) while one carried two compound heterozygous mutations in *USH2A* (NM_206933.2) gene (Table 2).

As for point number 3), we were able to identify the simultaneous presence of mutations in both *USH2A* and *EYA4* genes in Family 23, an Italian family apparently affected by autosomal dominant NSHL. WES revealed the presence of two compound heterozygous mutations in *USH2A* (NM_206933.2) in the proband (i.e., c.11864G > A, p.(Trp3955 *) and c.2276G > T, p.(Cys759Phe)) in addition to a stop gain variant in *EYA4* (NM_004100.4), i.e., c.714C > A, p.(Tyr238 *), segregating in the other affected family members (i.e., the proband’s mother, the maternal uncle and the maternal grandfather). Subsequently, the proband’s hearing thresholds appeared worse than those of her relatives, possibly due to the simultaneous presence of mutations in both *USH2A* and *EYA4A*.

**Figure 3:**
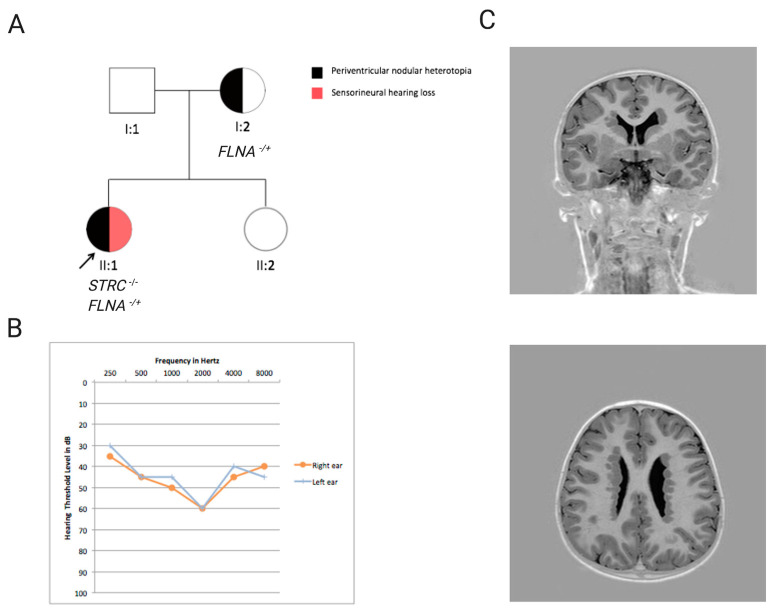
Pedigree, clinical and genetic features of Patient 84. (**A**) Pedigree of Patient 84, affected by both sensorineural hearing loss and periventricular nodular heterotopia. (**B**) Audiometric features of the affected individual, displayed as audiograms (air conduction). The thresholds of the right and left ears are shown. (**C**) Axial (coronal) scan IR T1-weighted. Bilateral periventricular nodules of grey matter are seen immediately deep to the ependymal layer of the bodies of both lateral ventricles.

In other cases, WES revealed the presence of 4) multiple independent genetic conditions that were initially misinterpreted as a single syndrome. An example is Patient 84, who presented with sensorineural hearing loss and periventricular nodular heterotopia (Figure 3).

MLPA detected a homozygous deletion in the *STRC* gene explaining the HL phenotype but not the neurological one. The application of WES allowed to identify a heterozygous nonsense variant in the *FLNA* gene ((NM_001456.3), c.1159C > T p.(Gln387 *)), a gene known for being causative of periventricular nodular heterotopia in an X-linked dominant fashion [48]. The variant was inherited from the mother, whose MRI revealed foci of periventricular nodular heterotopia, confirming the identified allele’s pathogenic effect.

**Figure 4:**
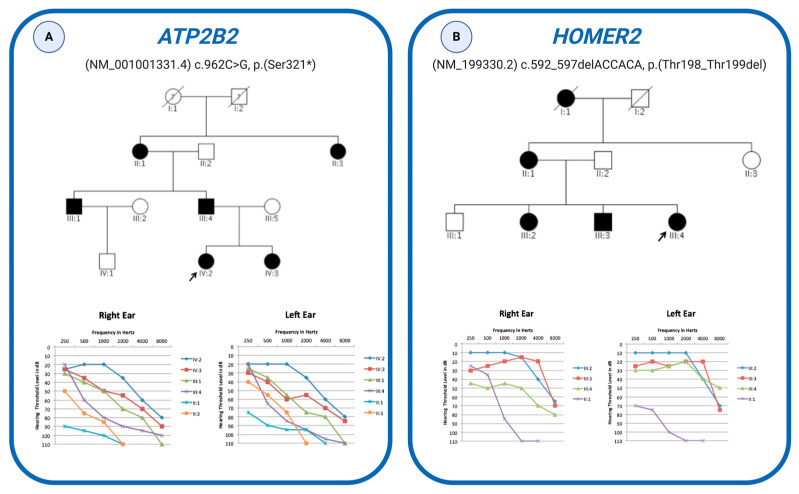
Pedigree and audiometric features of the families with novel variants in *ATP2B2* and *HOMER2* genes. (**A**) Pedigree of the family carrying a novel nonsense variant in the *ATP2B2* gene and audiometric features of the affected individuals. (**B**) Pedigree of the family carrying a novel deletion in the *HOMER2* gene and audiometric features of the affected individuals. Filled symbols represent affected individuals. Probands are indicated with an arrow. Individuals with Roman numeric labels were analyzed in this study. Audiometric features of the subjects are displayed as audiograms (air conduction). The thresholds of the right and left ears are shown.

WES also allowed 5) to detect novel variants in genes recently described as causative of NSHL, supporting their pathogenic role. An example is the case of Family 28, an Italian family presenting with a likely autosomal dominant NSHL (Figure 4A), where a novel nonsense variant in *ATP2B2* ((NM_001001331.4) c.962C > G, p.(Ser321 *) has been identified.

For many years *ATP2B2* has been described as a modifier of *CDH23* [49], and it has only recently been hypothesized that loss of function mutations in this gene cause autosomal dominant NSHL [50]. The identification of an additional ADNSHL family carrying a nonsense variant strengthens previous findings, confirming the pathogenic role of the ATP2B2 gene.

Another example is Family 32, an Italian family affected by NSHL (Figure 4B). WES revealed the presence of a novel heterozygous deletion in the *HOMER2* gene (NM_199330.2) (i.e., c.592_597delACCACA, p.(Thr198_Thr199del)) segregating within the family in an autosomal dominant fashion. To our knowledge, this represents the third independent NSHL family carrying a variant in this gene [12,51], definitely confirming its relevant role in the etiopathogenesis of hearing loss.

Finally, WES proved to be extremely efficient for the molecular diagnosis of clinically evident SHL. In particular, all the Usher patients (i.e., patients displaying HL and retinitis pigmentosa) were molecularly characterized, identifying homozygous or compound heterozygous mutations in *USH2A* and *MYO7A* genes (Table 2). Among the two suspected Alport patients enrolled in the study, one was a carrier of a variant in *COL4A3* ((NM_000091.5) c.3943C > T, p.(Pro1315Ser)) inherited from the affected father, while the second individual did not display any pathogenic mutation in all the genes known to be causative of such syndrome. Finally, a patient clinically diagnosed with Treacher–Collins syndrome carried a frameshift deletion in *TCOF1* ((NM_000356) c.4131_4135del, p.(K1380Efs * 11)) (Table 2).

## 4. Discussion

The definition of the molecular basis of HHL has always being a challenge for clinicians and geneticists. The development and application of a multi-step integrated strategy based on (1) an accurate clinical evaluation; (2) *GJB2/GJB6*/*MT-RNR1* screening; (3) MLPA; and (4) WES has proved to be a powerful approach for the molecular diagnosis of HHL patients.

**Figure 5:**
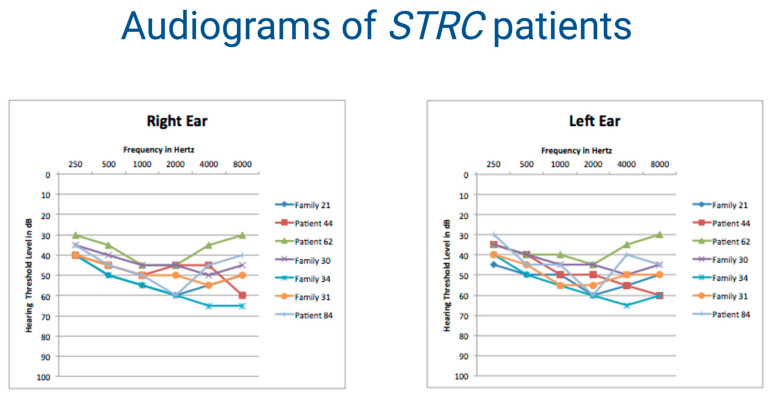
Audiometric features of the patients with *STRC* deletion/mutation. Audiograms of the patients with loss of function mutations or deletions of *STRC* gene display mild-to-moderate hearing loss. The thresholds of the right and left ears are shown.

Regarding NSHL, our data confirmed the relevant role of the *GJB2* gene responsible for 20% of cases, and identified *STRC* as the second major player in the Italian population, being causative of 6% of all NSHL patients. In this light, the application of MLPA, or other techniques able to identify CNVs, is becoming a crucial test for NSHL patients. Interestingly, in agreement with literature data [17], *STRC* deletions have been identified in patients revealing mild-to-moderate hearing loss (Figure 5), thus, supporting a possible genotype–phenotype correlation between these audiometric features and *STRC* loss.

On the other hand, no deletions in *GJB6* have been detected, and the A1555G mitochondrial mutation. These results, combined with the outcomes of the previous works [10,52] suggest that *GJB6* and *MT-RNR1* are not a common cause of NSHL in the Italian population, despite their relevant role in other areas of the world.

WES allowed the identification of the genetic cause of HHL in 86% of the SHL patients and 23% of the NSHL subjects, revealing some unexpected findings in the latter case. Indeed, 4% of patients received a molecular diagnosis of syndromic HL, despite the first clinical evaluation in favor of NSHL, and multiple genetic causes of the clinical phenotype were identified in two families, hampering the interpretation of the sequencing data.

These findings emphasize the usefulness of WES compared to other approaches, such as the use of comprehensive gene panels. In fact, WES allowed at once (a) an early diagnosis of the syndromic cases that do not already show all the clinical signs or symptoms, (b) the possibility of unveiling unrelated co-existing genetic conditions, (c) the identification of new deafness candidate genes not previously described or only detected as private mutation/gene of a single family worldwide, thus resulting in a cost and time-saving approach.

The results of the present study highlight the complexity of HL and, more importantly, have obvious clinical outcomes. A correct molecular diagnosis provides patients with significant prognostic value and relevant heritability information and influences their management, leading to tailored medical surveillance and different therapeutic options. Moreover, in the case of HHL, it has been demonstrated that knowing the gene involved in the disease can help predict the response to cochlear implantation. As an example, patients carrying mutations in the *GJB2* gene show an excellent response to cochlear implants. In contrast, those with mutations involving genes that affect the cochlear nerve itself gave worse post-implant performance [53]. Knowing this issue before implantation can help define expectations about post-implant auditory function.

Overall, all the examples discussed above point out the complexity of HHL (both syndromic and non-syndromic). When dealing with this phenotype, it is essential to be aware of the difficulties encountered in choosing the most effective approach to arrive at a correct molecular diagnosis. With this in mind, the collaboration between geneticists, clinicians, and otolaryngologists, who have an in-depth knowledge of the clinical features of hearing loss and the genes involved, is fundamental to achieve the ultimate goal of unraveling the genetic bases of HHL and improving the lives of patients.

## Figures and Tables

**Table 1:** List of patients positive for *GJB2* gene with indication of the identified mutations.

Patient ID	Gene	Variant 1	Variant 2	Status
Patient 1	*GJB2*	c.35delG, p.(Gly12Valfs * 2)	c.-27C > T	compound heterozygous
Patient 2		c.35delG, p.(Gly12Valfs * 2)	c.229T > C, p.(Trp77Arg)	compound heterozygous
Patient 3		c.35delG, p.(Gly12Valfs * 2)	c.35delG, p.(Gly12Valfs * 2)	homozygous
Patient 4		c.35delG, p.(Gly12Valfs * 2)	c.139G > T p.(Glu47 *)	compound heterozygous
Patient 5		c.35delG, p.(Gly12Valfs * 2)	c.358_360delGAG, p.(Glu120del)	compound heterozygous
Patient 6		c.35delG, p.(Gly12Valfs * 2)	c.-23 + 1G > A	compound heterozygous
Patient 7		c.35delG, p.(Gly12Valfs * 2)	c.71G > A, p.(Glu24 *)	compound heterozygous
Patient 8		c.35delG, p.(Gly12Valfs * 2)	c.71G > A, p.(Glu24 *)	compound heterozygous
Patient 9		c.35delG, p.(Gly12Valfs * 2)	c.35delG, p.(Gly12Valfs * 2)	homozygous
Patient 10		c.35delG, p.(Gly12Valfs * 2)	c.35delG, p.(Gly12Valfs * 2)	homozygous
Patient 11		c.35delG, p.(Gly12Valfs * 2)	c.314_327del14, p.(Lys105Argfs * 5)	compound heterozygous
Patient 12		c.35delG, p.(Gly12Valfs * 2)	c.35delG, p.(Gly12Valfs * 2)	homozygous
Patient 13		c.35delG, p.(Gly12Valfs * 2)	c.95G > A, p.(Arg32His)	compound heterozygous
Patient 14		c.35delG, p.(Gly12Valfs * 2)	c.35delG, p.(Gly12Valfs * 2)	homozygous
Patient 15		c.59T > C, p.(Ile20Thr)	c.314_327del14, p.(Lys105Argfs * 5)	compound heterozygous
Patient 16		c.35delG, p.(Gly12Valfs * 2)	c.71G > A, p.(Glu24 * )	compound heterozygous
Patient 17		c.35delG, p.(Gly12Valfs * 2)	c.35delG, p.(Gly12Valfs * 2)	homozygous
Patient 18		c.35delG, p.(Gly12Valfs * 2)	c.139G > T p.(Glu47 *)	compound heterozygous
Patient 19		c.35delG, p.(Gly12Valfs * 2)	c.35delG, p.(Gly12Valfs * 2)	homozygous
Patient 20		c.35delG, p.(Gly12Valfs * 2)	c.35delG, p.(Gly12Valfs * 2)	homozygous
Patient 21		c.101T > C, p.(Met34Thr)	c.358_360delGAG, p.(Glu120del)	compound heterozygous
Patient 22		c.35delG, p.(Gly12Valfs * 2)	c.35delG, p.(Gly12Valfs * 2)	homozygous
Patient 23		c.35delG, p.(Gly12Valfs * 2)	c.35delG, p.(Gly12Valfs * 2)	homozygous
Patient 24		c.35delG, p.(Gly12Valfs * 2)	c.358_360delGAG, p.(Glu120del)	compound heterozygous
Patient 25		c.35delG, p.(Gly12Valfs * 2)	c.35delG, p.(Gly12Valfs * 2)	homozygous

**Table 2:** List of likely causative variants identified by WES and Multiplex Ligation Probe Amplification (MLPA). All variants have been classified based on their frequency reported in Genome Aggregation Database (gnomAD, http://gnomad.broadinstitute.org/) and their pathogenicity. In particular, the following tools have been used: CADD PHRED (Pathogenicity score: > 10 predicted to be deleterious), GERP++_RS (higher number is more conserved), and Polyphen-2 (D: Probably damaging, P: possibly damaging; B: benign), SIFT (D: deleterious; T: tolerated), MutationTaster (A (disease_causing_automatic); D (disease_causing); N (polymorphism); P (polymorphism automatic)). NA = not available; hom = homozygous; het = heterozygous.

Family ID	Gene	cDNA Change	Protein Change	dbSNP	gnomAD_ALL	CADD PHRED	GERP ++_RS	Polyphen-2	SIFT	Mutation Taster	References
Family 3	*OTOA (NM_144672.3)*	entire gene deletion (hom)	NA	NA	NA	NA	NA	NA	NA	NA	Shearer et al., (2014) Genome Med [30]
Family 4	*COL11A2 (NM_080680.2)*	c.3100C > T (het)	p.(Arg1034Cys)	rs121912947	NA	32	2.95	D	D	D	McGuirt et al., (1999) Nat Genet [31]
Patient 41	*MYO7A (NM_000260.3)*	c.6236G > A (hom)	p.(Arg2079Gln)	rs765083332	0.00004188	25.5	4.24	P	T	D	NA
Patient 42	*ADGRV1 (NM_032119.3)*	c.10084C > T (het)	p.(Gln3362 *)	NA	NA	42	3.02	NA	NA	A	NA
		c.13655dupT (het)	p.(Asn4553Glufs * 18)	rs765376986	NA	NA	NA	NA	NA	NA	NA
Patient 44	*STRC (NM_153700.2)*	entire gene deletion (hom)	NA	NA	NA	NA	NA	NA	NA	NA	Vona et al.,(2015) Clin Genet [32]
Patient 45	*GJB1 (NM_000166.5)*	c.790C > T (het)	p.(Arg264Cys)	rs587777879	0.00005714	25.8	4.9	D	D	D	Numakura et al., (2002) Hum Mutat [33]
Patient 51	*CLDN14 (NM_144492.2)*	c.301G > A (hom)	p.(Gly101Arg)	rs74315438	0.00004302	26.5	5.42	D	D	D	Wattenhofer et al.,(2005) Hum Mutat [34]
Family 6	*SMPX (NM_014332.2)*	c.45+1G > A (hemizygous)	NA	NA	NA	24.4	4.9	NA	NA	D	NA
Family 7	*SIX1 (NM_005982.3)*	c.397_399delGAG (het)	p.(Glu133del)	rs80356460	NA	NA	NA	NA	NA	NA	Ruf et al., (2004) Proc Natl Acad Sci U S A [35]
Family 8	*KARS (NM_001130089.1)*	c.1423C > T (het)	p.(Leu475Phe)	NA	NA	28.2	5.91	P	T	D	NA
		c.1570T > C (het)	p.(Cys524Arg)	NA	NA	26.9	5.81	D	D	D	NA
Patient 57	*OTOG (NM_001277269.1)*	c.2500C > T (hom)	p.(Gln834 *)	rs554847663	0.0004274	37	2.01	NA	NA	D	Sheppard et al.,(2018) Genet Med [36]
Family 10	*PAX3 (NM_181457.3)*	c.220C > T (het)	p.(Arg74Cys)	NA	NA	35	5.24	D	D	D	Lenarduzzi et al., (2019) Hear Res [37]
Patient 62	*STRC (NM_153700.2)*	entire gene deletion (hom)	NA	NA	NA	NA	NA	NA	NA	NA	Vona et al.,(2015) Clin Genet [32]
Family 13	*ESPN (NM_031475.2)*	entire gene deletion (hom)	NA	NA	NA	NA	NA	NA	NA	NA	NA
Family 14	*MYO6 (NM_004999.3)*	c.1525delG (het)	p.(Val509Trpfs * 7)	NA	NA	NA	NA	NA	NA	D	NA
Family 15	*OTOF (NM_194248.2)*	c.2891C > A (hom)	p.(Ala964Glu)	rs201329629	NA	32	5.41	D	D	D	Rodriguez-Ballesteros et al. (2008) Hum Mutat [38]
Patient 65	*ADGRV1 (NM_032119.3)*	c.4378G>A (het)	p.(Gly1460Ser)	rs1303930496	0.00001248	32	5.53	D	D	D	Magliulo et al.,(2017) Otolaryngol Head Neck Surg [39]
		c.13655dupT (het)	p.(Asn4553Glufs * 18)	rs765376986	NA	NA	NA	NA	NA	NA	NA
Family 18	*DIAPH1 (NM_005219.4)*	c.3556delC (het)	p.(Leu1186Serfs * 2)	NA	NA	NA	NA	NA	NA	D	NA
Patient 70	*GATA3 (NM_002051.2)*	c.925-1G > T (het, de novo)	NA	NA	NA	25.9	5.3	NA	NA	NA	NA
Family 20	*TECTA (NM_005422.2)*	c.3841T > C (het)	p.(Cys1281Arg)	NA	NA	17.03	5.76	T	D	D	NA
Family 21	*STRC (NM_153700.2)*	entire gene deletion (hom)	NA	NA	NA	NA	NA	NA	NA	NA	Vona et al.,(2015) Clin Genet [32]
Family 23	*USH2A (NM_206933.2)*	c.2276G > T (het)	p.(Cys759Phe)	rs80338902	0.000947	33	5.79	D	D	D	Rivolta et al.,(2000) Am J Hum Genet [40]
		c.11864G > A (het)	p.(Trp3955 *)	rs111033364	0.000119	51	5.53	NA	D	D	van Wijk et al., (2004) Am J Hum Genet [41]
	*EYA4 (NM_004100.4)*	c.714C > A (het)	p.(Tyr238 *)	rs1264401894	0.000004	37	4.77	NA	D	D	NA
Family 24	*WFS1 (NM_006005.3)*	c.2567C > A (het)	p.(Pro856His)	NA	NA	23.7	4.82	D	D	D	NA
Family 26	*MYO6 (NM_004999.3)*	c.613C > T (het)	p.(Arg205 *)	rs557441143	0.00000398	37	3.31	NA	NA	A	Choi ET AL.,(2013) PLoS One 8 [42]
Family 27	*COL2A1 (NM_001844.5)*	c.4201G > C (het)	p.(Asp1401His)	NA	NA	19.9	5.06	D	D	D	NA
Family 28	*ATP2B2 (NM_001001331.4)*	c.962C > G (het)	p.(Ser321 *)	NA	NA	42	5.34	NA	T	A	NA
Patient 79	*LARS2*	4 Kb gene deletion (hom)	NA	NA	NA	NA	NA	NA	NA	NA	NA
Family 29	*PDZD7 (NM_024895.4)*	c.166dupC (het)	p.(Arg56Profs * 24)	rs587776894	NA	NA	NA	NA	NA	NA	Ebermann et al.,(2010) J Clin Invest [43]
		c.305G > A (het)	p.(Arg102His)	rs760825921	0.00001061	34	5.07	D	D	D	NA
Family 30	*STRC (NM_153700.2)*	entire gene deletion (hom)	NA	NA	NA	NA	NA	NA	NA	NA	Vona et al.,(2015) Clin Genet [32]
Family 31	*STRC (NM_153700.2)*	entire gene deletion (hom)	NA	NA	NA	NA	NA	NA	NA	NA	Vona et al.,(2015) Clin Genet [32]
Family 32	*HOMER2 (NM_199330.2)*	c.592_597delACCACA (het)	p.(Thr198_Thr199del)	NA	NA	NA	NA	NA	NA	P	NA
Patient 84	*STRC (NM_153700.2)*	entire gene deletion (hom)	NA	NA	NA	NA	NA	NA	NA	NA	Vona et al.,(2015) Clin Genet [32]
Family 34	*STRC (NM_153700.2)*	c.4057C > T (hom)	p.(Gln1353 *)	rs774312182	0.00006374	37	4.16	NA	NA	D	Shearer et al., (2010) Proc Natl Acad Sci U S A [44]
Patient 86	*TCOF1 (NM_001135243.1)*	c.4362_4366del (het)	p.(Lys1457Glufs * 11)	NA	NA	NA	NA	NA	NA	NA	NA
Family 35	*COL4A3 (NM_000091.4)*	c.3943C > T (het)	p.(Pro1315Ser)	rs760703010	0.00002793	25	5.57	D	D	D	NA
Patient 87	*USH2A (NM_206933.2)*	c.11864G > A (hom)	p.(Trp3955 *)	rs111033364	0.000119	51	5.53	NA	D	A	van Wijk et al., (2004) Am J Hum Genet [41]
Patient 88	*USH2A (NM_206933.2)*	c.4933G > T (hom)	p.(Gly1645 *)	NA	NA	38	3.86	NA	NA	A	Sloan-Heggen et al.,(2016) Hum Genet [45]
Patient 89	*USH2A (NM_206933.2)*	c.2035G > T (het)	p.(Gly679 *)	NA	NA	38	5.26	NA	NA	A	NA
		c.11864G > A (het)	p.(Trp3955 *)	rs111033364	0.000119	51	5.53	NA	D	A	van Wijk et al., (2004) Am J Hum Genet [41]
Patient 90	*MYO7A (NM_000260.3)*	c.735G > A (het)	(p.Gln245Gln)	NA	NA	NA	NA	NA	NA	A	Atik et al.,(2015) PLoS One 10 [46]
		c.1834_1836delAGC (het)	p.(Ser612del)	NA	NA	NA	NA	NA	NA	D	NA
